# Editorial: Future directions of active lifestyle promotion in community-dwelling older adults

**DOI:** 10.3389/fpubh.2024.1372316

**Published:** 2024-02-06

**Authors:** Johan de Jong, Fons van der Lucht, Afroditi Stathi

**Affiliations:** ^1^School of Sports Studies, Hanze University of Applied Sciences, Groningen, Netherlands; ^2^Center for Human Movement Sciences, University Medical Center Groningen, University of Groningen, Groningen, Netherlands; ^3^School of Health, Hanze University of Applied Sciences, Groningen, Netherlands; ^4^National Institute for Public Health and the Environment, Bilthoven, Netherlands; ^5^School of Sport, Exercise and Rehabilitation Sciences, College of Life and Environmental Sciences, University of Birmingham, Birmingham, United Kingdom

**Keywords:** active lifestyle, older adults, prevention, innovation, health

## Call for prevention and innovation

Future projections demonstrate that the number of people aged 65 years and older will double in the next 25 years. By 2050, one third of the European population will consist of older adults (1, 2). An increasing proportion of this group will live longer, managing health problems that have physical, social, and mental impact. These developments result in a higher overall societal burden and an unsustainable rise in health and social care costs. More health care employees will be needed to deliver the required levels of primary and secondary care. This adds considerable demand on financial resources for healthcare systems which already report a tremendous shortage of health professionals. A key strategy to cope with these challenges is to focus more on prevention and innovation regarding health promotion in older adults.

There is ample evidence about the impact of physical activity on the physical, mental and social wellbeing of older adults (3). An active lifestyle can lead to better vitality, reduction in the risk of falling, frailty and compression of the ill-health years toward the end of the lifespan (4, 5). For successfully promoting and implementing of an active lifestyle, new and innovative public health approaches are needed encompassing the entire prevention spectrum from broad universal to selective tailored forms of prevention for the community dwelling older adults. For that, a broader use of ecological models focusing on the interaction between older adults and their environmental contexts should be promoted (6, 7). Further, new ways to actively engage older adults should be tested and applied to achieve better compliance to interventions resulting in more positive health impact.

This special issue consists of eight articles which can be clustered in two main parts regarding the future directions of active lifestyle promotion in community-dwelling older adults. The first part describes the needs of older adults and health professionals/experts regarding health promotion, the determinants and factors of influence on health-related indicators in different countries. The second part provides two examples of good practice via (a) the active engagement of older adults in fall prevention programs and (b) the successful impact of a comprehensive group-based, exercise and behavioral maintenance programme targeting older people with mobility limitations.

## Factors, determinants and needs of active lifestyle promotion in older adults

In their explorative study among Iranian older adults and geriatric experts, Ayoubi-Mahani et al. revealed personal, managerial, and educational needs for active aging which could assist policymakers and geriatric experts to promote and meet active aging needs successfully.

Chen et al. explored the factors related to volunteering participation of older adults and their impact on successful aging in East Taiwan. Results confirmed that the motivation and expectation of middle-aged and older adults to participate in volunteering affected their continuous participation behavior and successful aging status through satisfaction.

Tan et al. investigated whether neighborhood socioeconomic status (SES) influenced activities of daily living/instrumental activities of daily living (ADL/IADL) in Chinese older adults mediated by the neighborhood built and social environment. Their main findings showed that neighborhood SES was significantly correlated with ADL/IADL mediated by the neighborhood environment. Improving the ADL/IADL status of older adults residing in low SES neighborhoods requires enhancing the built and social environment by provisioning neighborhood resources like exercise facilities, supermarkets, and neighborhoods activities (e.g., painting, dancing).

Dupré et al. in the PROOF cohort study, which started with 1,011 French 65 year older adults in 2001 (60% men), found a dose–response between the dose of light Intensity Physical Activity (LPA), moderate to vigorous physical activity, sedentary behavior, and risk of mortality. Their findings support the inclusion of LPA (e.g., walking at own pace) in future physical activity guidelines.

Costa et al. assessed the active aging awareness of older adults in Portugal and their levels of overall wellbeing and examined social and health-related factors associated with wellbeing. Their results revealed that a range of social (e.g., education level, financial situation) and health-related factors (e.g., illnesses, doctor visit) are associated with wellbeing and active aging awareness, and also with the differences reported among different SES groups in mainland Portugal. This emphasizes the importance to address social inequalities in active aging efforts, which are not necessarily uncovered when only considering actual active aging measures.

Jia et al. investigated the role of non-cognitive skills (e.g., personality, self-esteem, and emotions) in the process of reemployment of retirees in China. The results show that non-cognitive skills are related with Chinese retirees seeking reemployment. This relationship is stronger for male retirees, people who live in a rural household, and people who are of lower age and education level.

## Engagement and effects of active lifestyle interventions

In their exploratory qualitative study, Scherpenseel van et al. aimed to identify promising strategies that promote participation in fall prevention programs for Dutch community-dwelling older adults. Key proposed strategies were: (1) reframing aging and fall prevention into a life course perspective about falls, and avoiding confronting words; (2) informing about holistic benefits like living independently for longer. These insights could help in improving enrollment and maintenance of participation in fall prevention programs for older adults.

The REtirement in ACTion (REACT) study by Ladlow et al. investigated the effectiveness of the successful exercise intervention on individual markers of physical function in English older adults at 6-and 12-months. The REACT exercise program provides local, regional and national service providers with an effective solution to increase muscle strength and balance in older adults at risk of mobility disability.

## Perspectives

In the light of the current and foreseen aging of the global population and the societal burden in terms of the cumulative rise in health and social care costs and human resource challenge of quantity and quality of health care professionals, this Research Topic stressed the urgent need for more attention for prevention and innovative solutions toward Active Lifestyle. Since this is a global wicked problem, the included articles provide more insights in factors, determinants, needs, engagement processes, and effects of successful interventions from different countries and cultures. The underrepresentation of concrete interventions and aspects such as relevant technology for an active lifestyle in older adults in this issue, stress the need for further action.

## Author contributions

JJ: Writing—original draft, Writing—review & editing. FL: Writing—review & editing. AS: Writing—review & editing.
